# Brain Derived Exosomes Are a Double-Edged Sword in Alzheimer’s Disease

**DOI:** 10.3389/fnmol.2020.00079

**Published:** 2020-05-29

**Authors:** Zhiqi Song, Yanfeng Xu, Wei Deng, Ling Zhang, Hua Zhu, Pin Yu, Yajin Qu, Wenjie Zhao, Yunlin Han, Chuan Qin

**Affiliations:** Key Laboratory of Human Disease Comparative Medicine, Chinese Ministry of Health, Beijing Key Laboratory for Animal Models of Emerging and Remerging Infectious Diseases, Institute of Laboratory Animal Sciences, Chinese Academy of Medical Sciences, Comparative Medicine Center, Peking Union Medical College, Beijing, China

**Keywords:** brain derived exosomes, Alzheimer’s disease, cell-to-cell communication, biomarker, extracellular vesicles

## Abstract

Brain derived exosomes (BDEs) are extracellular nanovesicles that are collectively released by all cell lineages of the central nervous system and contain cargo from their original cells. They are emerging as key mediators of communication and waste management among neurons, glial cells and connective tissue during both physiological and pathological conditions in the brain. We review the rapidly growing frontier of BDEs biology in recent years including the involvement of exosomes in neuronal development, maintenance and communication through their multiple signaling functions. Particularly, we highlight the important role of exosomes in Alzheimer’s disease (AD), both as a pathogenic agent and as a disease biomarker. Our understanding of such unique nanovesicles may offer not only answers about the (patho) physiological course in AD and associated neurodegenerative diseases but also ideal methods to develop these vesicles as vehicles for drug delivery or as tools to monitor brain diseases in a non-invasive manner because crossing the blood brain barrier is an inherent capability of exosomes. BDEs have potential as biomarkers and as therapeutic tools for AD and related brain disorders in the near future.

## Introduction

Exosomes are small extracellular nano-sized vesicles between 30 and 150 nm in diameter (DeLeo and Ikezu, 2018) that were first described in the 1980s (Johnstone et al., 1987). They consist of one type of EVs and are categorized on the basis of their biogenesis pathways (Pan and Johnstone, 1983). Since then, exosomes have been isolated from nearly all mammalian cell types, including cells in the CNS such as neurons, astrocytes, oligodendrocytes, microglia, and Schwann cells, as well as endothelial cells (Faure et al., 2006; Sharma et al., 2013). Exosomes released from the nervous system are collectively called BDEs.

**FIGURE 1:**
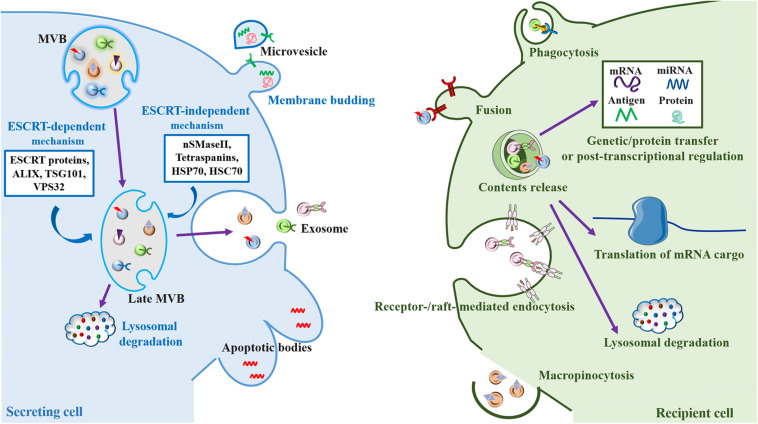
Biogenesis and cell-to-cell communication of extracellular vesicles (EV), especially exosomes. Exosomes are formed by the inward budding of the multivesicular body (MVB) membrane, which is formed by invagination of the endosomal membrane. Early endosomes go through transition to late endosomes and are further burdened to form late MVBs containing intraluminal vesicles (ILVs) (Colombo et al., 2014). Cargo sorting into exosomes involves the endosomal sorting complex required for transport (ESCRT)-dependent mechanism, which are managed by ESCRT proteins (ESCRT-0, I, II, and III) and their accessories (ALIX, TSG101, and VPS32) (Hurley, 2010; van Niel et al., 2018), and ESCRT-independent mechanism, which are mediated by neutral sphingomyelinase 2 (nSMaseII), tetraspanins, and the chaperone heat shock proteins (HSP70 and HSC70), can develop ILVs (Malla et al., 2018). ILVs have two fates, either fusing with the lysosome resulting in the degradation of the ILVs and their contents for recycling as an endolysosomal pathway (Klumperman and Raposo, 2014), or fusing with the plasma membrane where they are released into the extracellular space as exosomes through a secretory pathway (Heijnen et al., 1999). Microvesicles are formed directly by outward budding of the plasma membrane, a course which is managed by the ESCRT components and ADP ribosylation factor 6 (ARF6), some small GTPases, lipids, and Ca^2+^-dependent enzymatic machineries (Thompson et al., 2016). Apoptotic bodies are the largest of the EVs. They “bleb” off the cell membrane and contain material from cells undergoing apoptosis, which are typically engulfed by macrophages (Thompson et al., 2016). After release into the extracellular space, exosomes can be internalized by recipient cells mediated by the interaction of various exosomal surface proteins and cellular receptors via several mechanisms including phagocytosis, plasma membrane fusion, macropinocytosis and endocytosis (McKelvey et al., 2015). The contents of exosomes can effectively influence cellular processes through taking part in genetic/protein transfer, transcriptional regulation or post-transcriptional regulation. Alternatively, exosomes can be further fused with the lysosomes for degradation (Saeedi et al., 2019; You and Ikezu, 2019).

To form MVBs with ILVs, early endosomes undergo inward budding (Colombo et al., 2014). Then, ILVs are released into the extracellular environment as exosomes via fusion of MVBs with the plasma membrane (Heijnen et al., 1999; Mulcahy et al., 2014). Alternatively, exosomal formation can be regulated by sphingolipids, ceramides and tetraspanins (Trajkovic et al., 2008; Stuffers et al., 2009; van Niel et al., 2011). Otherwise, MVBs can fuse with the lysosomal membrane, resulting in degradation of ILVs and recycling of their content (Stuffers et al., 2009; Klumperman and Raposo, 2014). As a result of their origin and multifarious molecular cargo, including but not limited to gDNA, mRNA, other non-coding RNAs, lipids and proteins (Yokoi et al., 2019), the molecular species and relative amounts in exosomes are highly heterogeneous and complex in composition. Exosomes can randomly or selectively exhibit great variety depending on their membranes, cytosolic proteins, and nucleic acids compared with the cells that release them. Based on the proteomic and other comprehensive analyses, the heterogeneity of exosomes is conceptualized on the basis of their size, content (cargo), functional impact on recipient cells and cell of origin (source) (Kalluri and LeBleu, 2020). It is becoming increasingly clear that exosomes have specialized functions and play a key role in coagulation, intercellular signaling and waste management (van der Pol et al., 2012; Figure 1).

During the formation process, exosomes are comprised of enriched endosome-associated components such as flotillins and annexins (van Niel et al., 2006), ALIX, the ESCRT component, tumor susceptibility gene 101 protein (TSG101) (Lotvall et al., 2014) and lipid rafts, including cholesterol, sphingomyelin and ceramide (DeLeo and Ikezu, 2018). Moreover, membrane proteins, including tetraspanins such as abundant CD9, CD63, and CD81, that are considered as markers for exosomes and play important roles in the biogenesis of endosomes or MVBs (Lotvall et al., 2014; Kowal et al., 2016). Depending on the cell type from which they are secreted, exosome vesicles themselves also hold particular and varied types of markers that contribute to identifying their origins (Lotvall et al., 2014; Kowal et al., 2016). For example, neuronal L1 cell adhesion molecule (L1CAM) can be used as a biomarker for isolating BDEs (Cicognola et al., 2019).

Once the exosomes are secreted, they can be internalized from the extracellular space by recipient cells through several mechanisms including phagocytosis, micropinocytosis, endocytosis, and plasma membrane fusion (McKelvey et al., 2015; Figure 1). On the other hand, exosomes carrying multiple cargo with valuable biological information can also be released into most bodily fluids such as plasma, cerebrospinal fluid, urine, saliva, amniotic fluid, colostrum, breast milk, synovial fluid, semen and pleural ascites, not only in normal tissues (Corrado et al., 2013; McKelvey et al., 2015) but also in aberrant bodies such as tumors (Camussi et al., 2011; Rak and Guha, 2012). For the above reasons, exosomes play key roles in the management of normal physiological environments, such as immune surveillance (Rajendran et al., 2006), stem cell maintenance (Ratajczak et al., 2006), tissue repair (Gatti et al., 2011), and blood coagulation (Del et al., 2005), as well as in the pathological processes associated with several diseases (Liu et al., 2019), including neurodegenerative diseases such as AD (Bellingham et al., 2012; Watson et al., 2019) and PD (Emmanouilidou et al., 2010).

Given the characteristic described above, exosomes and their constituents represent a novel class of therapeutic targets and such features also give them advantages as biomarkers to distinguish healthy and disease states (Emmanouilidou et al., 2010) and for prognosis prediction and therapy for diseases. Moreover, recent studies have demonstrated that exosomes may also be directly considered as potential therapeutic agents. For instance, mesenchymal stem cell-derived exosomes have been used in tissue regeneration (Lai et al., 2011; Timmers et al., 2011) and tumor antigen-pulsed dendritic cell-derived exosomes have been developed for cancer immune response modulation (Zitvogel et al., 1998). Remarkably, exosomes have an efficient capability to cross the BBB (Chen et al., 2016). In particular, exosomes isolated from CSF are rich in proteins that originate from the brain such as neuron-specific markers, microglial markers (CD11b and CD45) and Apo-E (Chiasserini et al., 2014), making them potential novel drug delivery vehicles for treating nervous system diseases. Here, we mainly discuss and summarize the role of BDEs in normal biological processes in the CNS as well as the aberrant pathological state of AD and focus on explaining how exosomes can be targeted or directly exploited as biomarkers or therapeutics in AD.

## Physiological Roles of BDEs in the CNS

**FIGURE 2:**
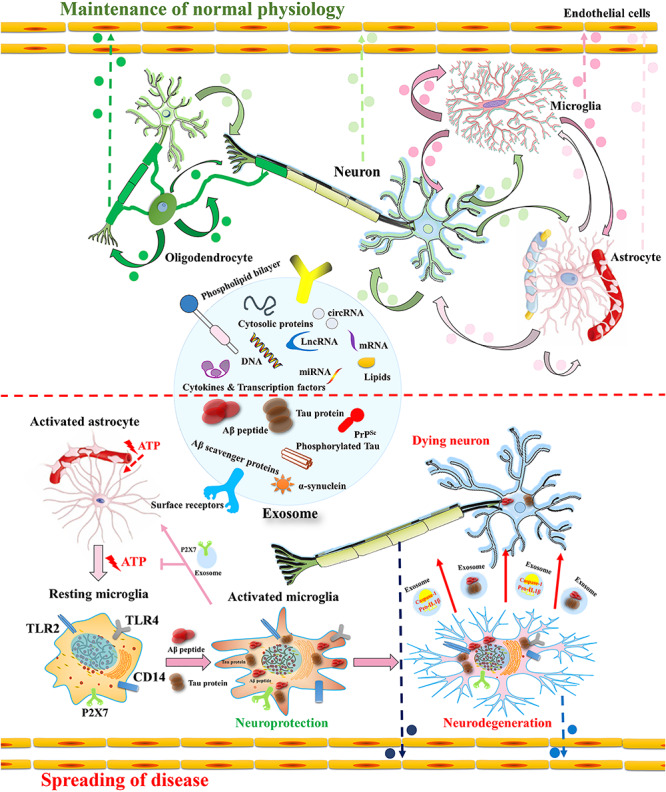
Intercellular communication of BDEs in the CNS in (patho) physiological course in AD. Exosomes secreted from oligodendrocytes, microglia, astrocytes and mesenchymal stem cells includes host cell derived cytosolic proteins, cytokines and transcription factors, Aβ scavenger enzymes, Aβ (Saeedi et al., 2019; You and Ikezu, 2019) and tau protein (DeLeo and Ikezu, 2018) along with nucleic acids (DNA, mRNA, miRNA and/or lncRNA and/or cirRNA) (Camussi et al., 2011; Lee et al., 2012; Yokoi et al., 2019). They afford positive as well as negative effect on the neurons depending on the cargo they carry. In the physiological condition, exosomes benefit to the reciprocal communication between neural cells (e.g., neuron-glia interaction), synaptic plasticity, neuronal development and neuroimmune communication. In the early stage of AD, the microglia activation by Aβ has neuroprotective effect because it induces phagocytosis and Aβ clearance (Bolmont et al., 2008; Hickman et al., 2008). The microglia stimulation by astrocyte released ATP induces the secretion of exosomes including P2X7 receptor as a defensive strategy to escape astrocyte signaling (Bolmont et al., 2008; Hickman et al., 2008). In the latter stage of AD, microglia secrete exosomes containing pro-IL1β, active caspase-1 and soluble toxic Aβ that have detrimental effects on neurons (Bianco et al., 2005; Trotta et al., 2018). Neurodegenerative associated proteins such as Aβ, Tau, prions (Faure et al., 2006) and α-synuclein (Emmanouilidou et al., 2010) can also be derived from neural derived exosomes, leading to the spread of protein aggregate seeds and disease progression. In addition, these exosomes could be exported via blood-brain barrier as circulatory EVs, which can be used for disease-specific biomarkers, even future for therapeutic researches (Thompson et al., 2016).

Exosomes exert their effects on essential biological processes throughout the body including the CNS by different mechanisms. These mechanisms include cell surface receptors activation through direct binding to lipid ligands and proteins, exosomal membrane fusion contents with the recipient cell plasma membrane and effectors delivery. Some of these effectors are oncogenes, transcription factors, small and large non-coding regulatory RNAs (such as miRNAs) and mRNAs, as well as infectious particles such as amyloid-β (Aβ)−derived (Bellingham et al., 2012) and α-synuclein (Emmanouilidou et al., 2010) into recipient cells (Camussi et al., 2011; Lee et al., 2012; Figure 1). In this manner, exosomes participate in the maintenance of normal physiology (Figure 2).

Particularly, in the CNS, exosomes can be released from virtually all cell types, including various types of neurons, astrocytes, oligodendrocytes, microglia, and Schwann cells, as well as endothelial cells (Faure et al., 2006; Sharma et al., 2013). EVs carry and release multiple molecules related to neuronal function and neurotransmission in the brain, which is beneficial for the reciprocal communication between neural cells (e.g., neuron−glia interactions), synaptic plasticity, neuronal development, and neuroimmune communication (Fruhbeis et al., 2013a; Rajendran et al., 2014; Iraci et al., 2016; Table 1).

**TABLE 1:** Exosome is a double-edged sword in nervous system.

**Condition**	**Function**		**References**
Physiological roles	Neuron−glia communication	Neuron and microglia	Fruhbeis et al., 2013b
		Neuron and astrocytes	Men et al., 2019; Pascua-Maestro et al., 2019
		Neuron and oligodendrocytes	Kramer-Albers et al., 2007
Pathological roles in AD	Synaptic plasticity and neurotransmission		Chivet et al., 2012; Koniusz et al., 2016
	Enhancement of neuron protection		Guitart et al., 2016
	Improvement neuronal development		Drago et al., 2017
	Carry the two hallmarks of AD brains, Aβ, and hyperphosphorylated tau		Sardar et al., 2018
	The spread of oligomers and neurotoxicity		Hamlett et al., 2018
	Carry the synaptic proteins		Goetzl et al., 2016a
	Carry the ceramide and sphingosine-1-phosphate (S1P)		Yuyama et al., 2012; Dinkins et al., 2014
	Containing tyrosine phosphorylated insulin receptor substrate 1 (IRS1)		Kapogiannis et al., 2015

### Neuron-Glia Communication via Exosome Secretion

Neurons and glial cells (a class of cells that mainly includes microglia, astrocytes, and oligodendrocytes) orchestrate CNS homeostasis via numerous mechanisms of intercellular communication. Exosomes might regulate the physiological condition of the recipient cells and interactions between various neural cells. For example, upon activation of glutamatergic synapses, cortical neuron-derived exosomes are selectively delivered to neurons but not glial cells (Koniusz et al., 2016). Recent studies using CD63-GFP positive intraluminal vesicles as exosomal reporter in mice have demonstrated that exosomes participate in mediating neuron to astroglia communication in the CNS (Men et al., 2019). Furthermore, miRNAs (especially miR-124a) in exosomes isolated from neuron-conditioned medium possess excitatory amino acid transporter 2, a necessary mediator of glutamate uptake via the internalization of exosomes into astrocytes (Morel et al., 2013). However, exosomes also participate in reciprocal oligodendrocyte-neuron communication and transfer cargo from oligodendrocytes to neurons (Fruhbeis et al., 2013a). Although exosome-mediated communication and the manner by which exosomes select their recipient cells are largely unclear, emerging evidence suggests that exosomes serve as selectively important conveyers for neuron-neuron or neuron-glia interaction in the brain by transmitting genetic information, various bioactive proteins, and lipids.

### Synaptic Plasticity and Neurotransmission by the Release of Exosomes

Maintenance and improvement of synaptic connectivity in the adult brain are crucial for cognitive function. Neural synaptic plasticity is mediated not only by neuron-specific progression but also by glial cells, such as astrocytes and microglia (Morris et al., 2013). Under resting conditions, synaptic vesicles are reposited in the cytoplasm of the nerve terminal. Many synaptic vesicles stick on some specialized sites at the presynaptic plasma membrane named active zones. During incoming action potentials, exocytosis of synaptic vesicles confirm how much transmitter is released from nerve terminals (Jahn and Fasshauer, 2012). In addition to typical synaptic neurotransmission, signal transduction of neurons via the secretion of exosomes can induce a range of neurobiological functions including synaptic plasticity (Chivet et al., 2012; Koniusz et al., 2016). During management of synaptic plasticity, PC12 cell-derived exosomes can stimulate synaptic pruning through enhancement of the complement component 3 level in microglial MG6 cells (Bahrini et al., 2015). For instance, enhanced secretion of cortical neuron-derived exosomes containing neurotransmitter receptors leads to increased glutamatergic activity (Lachenal et al., 2011). Additionally, by managing the number of AMPA receptors for glutamate transmission, neuronal-derived exosomes might affect synaptic plasticity (Lachenal et al., 2011).

### Enhancement of Neuron Protection and Neuronal Development Through Exosomes

Although the understanding of astrocyte-neuron communication via exosomes remains unclear, evidence supports that it does occur, and this communication manner is required for neuronal cell survival (Figure 2; Pascua-Maestro et al., 2019; Luarte et al., 2020). Neuroprotective signaling is essential for neuronal growth and survival. PrP is a physiologically important receptor protein that protects against oxidative stress in the CNS. Protection of neurons through astrocyte-derived exosomes is dependent on astrocyte-derived exosomal PrP transport into neurons (Guitart et al., 2016). In addition, studies have demonstrated that microglia and oligodendrocyte-derived exosomes contribute to neuronal energy metabolism by transferring several enzymes involved in energy metabolism (Kramer-Albers et al., 2007; Drago et al., 2017). Thus, exosomes mediate several vital processes involved in normal brain function. Taken together, these findings suggest that exosome-mediated cell communication is emerging as a method of mediating neuron protection.

## Pathological Roles of BDEs in AD

Increased secretion of exosomes is generally thought to occur in response to stress or pathological conditions (Urbanelli et al., 2013; Saxton and Sabatini, 2017; Gill et al., 2018), such as hypoxia (King et al., 2012; Ophelders et al., 2016), alcoholism (Momen-Heravi et al., 2015), cisplatin- (Xiao et al., 2014), or irradiation-induced DNA damage (Lehmann et al., 2008), and oxidative stress (Atienzar-Aroca et al., 2016). Neuron-derived exosomes including particular proteins associated with neurodegenerative diseases can be secreted from the affected neurons (Watson et al., 2019). In the case of AD, there are two hallmarks of AD brains, Aβ and hyperphosphorylated tau, interact with specific endosomes and may contribute to exosome biogenesis in AD (Sardar et al., 2018). Additionally, exosomal proteins, such as flotillin-1 and Alix, were observed to accumulate around amyloid plaques in AD patients (Rajendran et al., 2006). The formation, secretion or uptake of exosomes plays a dual role in the spread of oligomers and neurotoxicity (Table 1).

### Exosomes Containing Aβ in AD

Amyloid β-protein is a C-terminal cleavage product of the transmembrane APP produced by β- and γ-secretase (Devi and Anandatheerthavarada, 2010; Miranda et al., 2018). AD is characterized by the presence of aggregates of pathologically misfolded proteins in the brain, including extracellular senile plaques mainly consisting of Aβ (Brunkhorst et al., 2014). Numerous reports have described these proteins and their substrates within exosomes of *in vitro* AD models and in exosomes derived from neurons of AD patients (Dinkins et al., 2014; Yuyama et al., 2015).

Although most AD cases are sporadic, there is a minority patients from mutations in the genes encoding APP or during sequential cleavages by β- and γ-secretase enzyme activities (Chow et al., 2010). *In vitro*, exosomes isolated from neuronal cell lines show that inducing AD mutations can increase sAPP protein β, sAPPα (Xiao et al., 2017; Sinha et al., 2018) and soluble Aβ1-42 (Eitan et al., 2016a). N2a cells expressing human APP with the autosomal dominant Swedish mutation contain Aβ peptides as well as the C-terminal fragments of APP have also shown increase of production of C-end terminal fragments (a byproduct of APP after β-secretase processing) (Laulagnier et al., 2018), β-secretase in released exosomes, and co-localization of β-secretase enzyme 1 with early exosome markers (Xiao et al., 2017). Vesicles released by Aβ-treated astrocytes contain the pro-apoptotic prostate apoptosis response 4 (PAR-4) protein and these vesicles cause PAR-4 associated apoptosis in naive cultures (Wang et al., 2012). Experiments involving the medium of neural cells expressing familial AD presenilin 1 mutations show that Aβ is associated with exosomes during their excretion process (Eitan et al., 2016b).

*In vivo*, rodent exosomes can contain Aβ, BACE1, and presenilin 1 and 2 (Sharples et al., 2008). Exosomes isolated from bodily fluids of AD mouse model, including blood, CSF and urine, display indicative increase in the C-terminal fragments of APP (Laulagnier et al., 2018; Miranda et al., 2018). Exosomes isolated from bodily fluids of AD patients exhibit a remarkable increase soluble Aβ1–42 in Abner et al. (2016); Winston et al. (2016), Hamlett et al. (2017, 2018). Amyloid plaques in AD brains also contain an exosome marker (Abner et al., 2016; Winston et al., 2016; Hamlett et al., 2017, 2018). The muskelin protein is involved in reorganization of the cytoskeleton and has been shown to be involved in the determination of either lysosomal degradation or exosome secretion of PrP (Heisler et al., 2018). Importantly, PrP is a receptor for Aβ and is reported to increase the pathogenicity of AD (Cohen et al., 2016). Muskelin may play a critical role in this type of amyloidosis.

The above data suggested the hypothesis that exosomes could seed Aβ aggregation (Dinkins et al., 2017). However, neuronal exosomes can also restrain Aβ oligomerization and accelerate Aβ fibril formation, facilitating microglia-mediated Aβ clearance *in vitro* (Yuyama et al., 2012). Moreover, in an AD mouse model, intracerebral loading of glycosphingolipid-enriched exosomes led to trapping and transporting Aβ into microglia, leading to a decrease in Aβ pathology (Yuyama et al., 2014). These results might explain why, at least under some circumstances, exosomes associated with Aβ have a physiological, neuroprotective function (Yuyama and Igarashi, 2017). It is also possible that in the brain as exosomes are secreted by various cell types (e.g., neurons, microglia, and astrocytes), they might exhibit contrary effects or the exosomal membranes might promote Aβ aggregation independent of protein-associated exosomal functions (e.g., Aβ degradation by exosomal insulin-degrading neprilysin or enzymes) (reviewed in Dinkins et al., 2017).

### Exosomes Containing Hyperphosphorylated Tau in AD

The gradual deposition of hyperphosphorylated tau protein within specific neurons is pivotal to the tauopathy of AD (Saman et al., 2014; Takeda, 2019). Under normal physiological conditions, incorporation of neuronal microtubule-associated protein tau for microtubule elongation is a crucial event of neuronal synapse formation and synaptic plasticity. Additionally, intracellular tau also participates in neurite outgrowth, axonal transport, chromosome stability, regulation the cellular transcriptome and the structural architecture of heterochromatin (for more details see the review by Sotiropoulos et al., 2017). Extracellular tau is also secreted into brain interstitial fluid (Yamada et al., 2011) and may contribute to some characteristics of sleep (Lucey et al., 2019). The above functions rely on the site-specific phosphorylation of tau (Kapitein and Hoogenraad, 2015) in normal condition. However, hyperphosphorylation and aggregation of the microtubule-associated tau protein into intracellular neurofibrillary tangles is one of the classical pathological hallmarks of advanced-stage AD (Johnson and Stoothoff, 2004; Martin et al., 2011).

During the progressive accumulation of neurofibrillary tangles, tau becomes hyperphosphorylated in neurons. Meanwhile, the cellular clearance machinery takes up tau for degradation and packaging in exosomes (Saman et al., 2012; Chesser et al., 2013; Perez et al., 2019). In an adeno-associated virus-based mouse model revealing rapid tau propagation, microglia help to spread tau through exosome release, and depletion of microglia or inhibition of exosome synthesis significantly decreases the propagation of tau *in vitro* and *in vivo* (Asai et al., 2015). In another mouse model of tauopathy, aggregated tau was isolated from and transmitted through brain exosomes (Asai et al., 2015). In these mice, exosomes that were isolated from the brains of tau transgenic rTg4510 mice containing human four-repeat tau with the P301L mutation accelerated pathological tau phosphorylation and oligomer formation (Polanco et al., 2016), indicating that neuronal exosomes containing human mutated tau are toxic to the recipient neurons *in vivo* (Baker et al., 2016). Recently, *BIN1* was found to support spreading of tau via exosome release in mice. Tau-containing exosomes isolated from the CSF of AD-affected individuals who contain *BIN1*-associated genetic variants in AD etiology showed seeding competence (Crotti et al., 2019). In human patients, compared with BDEs obtained from the plasma or serum of age-matched controls, BDEs from AD patients showed a 3-20-fold increase in tau phosphorylation at threonine 181 (p-T181-tau) and serine 396 (p-S396-tau) (Crotti et al., 2019). Moreover, compared with AD patients who had only been diagnosed with mild cognitive impairment, p-T181-tau levels were significantly higher in BDEs isolated from the plasma of later-stage AD patients (Winston et al., 2016), demonstrating either a dysfunction of the clearance ability or an increase in pathogenicity of exosomes in later disease states of AD. p-T181- and p-S396-tau were significantly decreased in BDEs of patients 1–10 years prior to their AD diagnosis (Fiandaca et al., 2015).

### Exosomes Containing Synaptic Proteins in AD

One consequence of AD is neuron loss and dysfunction. The levels of synaptic proteins, including synaptophysin, synaptotagmins, synaptobrevin, synaptopodin, Rab3A, GAP 43, and neurogranin, were decreased in the BDE cargo from the plasma of AD patients (Goetzl et al., 2016b). Additionally, low-density LRP 6, REST, heat shock factor protein 1, HSP, and AMPA receptor levels are also lower in BDEs from the plasma of AD patients (Goetzl et al., 2015, 2016b; Winston et al., 2016). Furthermore, neurexin 2α, GluA4-containing glutamate receptor, and neuroligin 1, essential proteins for long-term potentiation processes, were all significantly reduced in BDEs from the plasma of patients 6−11 years prior to AD diagnosis and, along with neuronal pentraxin 2, were all downregulated in BDEs (Goetzl et al., 2018, 2019). These proteins are all involved in normal homeostasis of neurons. Further research into these proteins in BDEs could be beneficial for the search for earlier biomarkers of AD.

### Ceramide and Sphingosine-1-Phosphate (S1P) in Exosomes in AD

Activated sphingolipids are signaling molecules that serve as intracellular second messengers and include ceramide, sphingosine, and their derivatives, 1-phosphates (C1P and S1P, respectively) (Czubowicz et al., 2019). Exosomes are sphingomyelin- and ceramide-enriched vesicles formed inside MVEs and then are secreted when the MVE membrane fuses with the plasmalemma. Exosomes can serve as a vehicle for the extracellular secretion and cell-to-cell transport of Aβ, α-synuclein and tau protein, possibly further facilitating the spread of toxic protein aggregation (Ngolab et al., 2017; Wang et al., 2017). S1P receptor (S1PR) signaling has been reported to participate in exosomal cargo sorting. Activity of the S1PR-mediated Rho family of GTPases is essential for this process and Gβγ inhibitors inhibit this activity (Kajimoto et al., 2018). The secretion of exosomes can be regulated by the activation of neutral sphingomyelin synthase 2 (SMS2) and sphingomyelinase 2 (nSMase2), demonstrating distinct functions for these enzymes in AD (Yuyama et al., 2012; Dinkins et al., 2014). Additionally, ceramide/sphingolipid shortage results in enhanced secretion of sAβPPα, the product of non-amyloidogenic cleavage. However, this shortage gives rise to increased secretion of Aβ42 at the same time, probably via regulation of raft-associated proteins, resulting in alteration of the α- *vs.*β-cleavage ratio (Yuyama et al., 2012; Dinkins et al., 2014). Enhanced endogenous ceramide and exogenous additional ceramide both elevate the Aβ level (Puglielli et al., 2003; Sawamura et al., 2004). The above research suggests the significance of the ceramide/sphingolipid levels in the process of AD.

### Exosomes Containing Other AD-Associated Proteins

Dysregulation of insulin by the CNS and peripheral hyperinsulinemia have been reported as other events highly associated with AD (de la Monte, 2009; de la Monte and Tong, 2014; Kim et al., 2015). A low tyrosine phosphorylated insulin receptor substrate 1 (IRS1) to serine phosphorylated IRS1 ratio is a characteristic of insulin dysregulation (Aguirre et al., 2002; Draznin, 2006) and is related to greater brain atrophy in AD patients (Mullins et al., 2017). BDEs isolated from the plasma of human AD patients have revealed an enhancement in serine phosphorylation in IRS1 (Kapogiannis et al., 2015). This study demonstrated that significant differences in the IRS1 levels were recognizable up to 10 years prior to clinical onset of AD, which suggests that proteins within BDEs that are involved in insulin disruption may potentially be useful biomarkers for clinical diagnosis.

Moreover, exosomes have the ability to spread toxic proteins through PrP activity (Hartmann et al., 2017). PrP is a cell surface-anchored protein that is highly related to AD pathology (Kellett and Hooper, 2009). Its pathological, misfolded form is associated with spongiform encephalopathy (Prusiner, 1982; Song et al., 2013). Studies in animal models of AD have demonstrated that the PrP receptor is essential for the cognitive impairment linked to Aβ (Gimbel et al., 2010). Additionally, growing evidence indicates that prion receptor on exosomes are capable of transmitting pathological substances (Fevrier et al., 2004; Hartmann et al., 2017). Aberrant autophagy is likely to play a role in this process (Abdulrahman et al., 2018). However, further research is needed to explore the potential mechanism connecting exosomes, AD pathogenesis, and autophagy.

## Exosomal MiRNA as a Diagnostic Biomarker for AD

Generally, the biomarkers used for AD diagnosis include the expression of Aβ and pTau (Abdulrahman et al., 2018), and methods such as neuropsychological testing and specialized brain imaging techniques have also been widely used for diagnosis of AD. Unfortunately, most AD patients are asymptomatic during the pre-clinical stages, which may last up to 17 years or longer (Villemagne et al., 2013). Therefore, it is important to exploit early diagnostics to confirm and treat individuals who are at risk before severe and irreversible neuronal pathology occurs.

MiRNAs are a family of 18–22 nt single-stranded RNAs that post-translationally communicate with and regulate the expression of mature mRNAs. Single upregulated miRNAs can target various mRNAs to decrease their expression and multiple miRNAs can target a single mRNA (Sethi and Lukiw, 2009; Sarkar et al., 2016). Studies have demonstrated that mRNA and miRNA species are present in exosomes. It is possible that some mRNA sequences are definitely targeted for secretion by these vesicles (Valadi et al., 2007; Van Giau and An, 2016). Exosomal miRNAs play essential roles in intercellular communication between cell membranes in the CNS and in disease progression. Exosomal miRNAs are also ideal targets for use as potential biomarkers in clinical diagnostics or therapies as they can be analyzed through neuronal exosomes in the patient’s body fluids (A). Indeed, some research has illustrated that proteins and miRNAs can be transferred from glia to axons (Skog et al., 2008). It is assumed that miRNA signaling can impact neurodegenerative diseases via the dysregulation of tau, leading to neurotoxicity. One study convincingly demonstrated that, in brain tissues obtained at autopsy from AD patients and from those with severe primary age-related tau pathology, the level of the highly conserved miRNA-219 was decreased in the brain (Santa-Maria et al., 2015). Several reports have illustrated that high expression of tissue-specific miRNAs in the brain, such as miR-9, miR-29a/b, miR-107, miR-124, miR-128, miR-134, and miR-137, may result in defective neuronal development (Sempere et al., 2004; Kawase Koga et al., 2009; Huang et al., 2010). In addition, other miRNAs are also abnormal in brain tissues during neurodegenerative processes. These specific miRNAs, including miR-132 and miR-212, are among the most robustly declining miRNAs in neurodegenerative diseases, including AD (Cogswell et al., 2008; Hebert et al., 2013; Wong et al., 2013; Lau et al., 2014), Huntington’s disease (Johnson et al., 2008; Packer et al., 2008), PD (Burgos et al., 2014), schizophrenia and bipolar disorders (Perkins et al., 2007; Kim et al., 2010) and frontotemporal dementia (Chen-Plotkin et al., 2012; Hebert et al., 2013).

Remarkably, miRNAs that were found to be greatly expressed in the brain were also detected in human body fluids such as the plasma, urine, and CSF. The levels of brain-enriched miRNAs including miR-9, miR-29a, miR-29b, and miR-137 have been found to be significantly decreased in plasma samples collected from AD patients (Geekiyanage et al., 2012). Both miR-128 and miR-134 were also examined in patients with mild cognitive impairment, which is an early stage of AD (Sheinerman et al., 2012). These miRNA biomarkers in the blood represents a clinical advantage for early disease diagnosis, but differential miRNA expression may not accurately rescue abnormal miRNA expression in the brain. The CSF represents a more relevant and suitable source for the diagnosis of CNS disorders such as AD (Alexandrov et al., 2012; Patz et al., 2013; Van Giau and An, 2016) because CSF is a clear biological fluid produced in the choroid plexus of the brain that circulates via the inner ventricular system, crosses the BBB, and is absorbed into the bloodstream. The levels of target candidate miRNAs such as miR-9, miR-146a, and miR-155 were shown to be greatly increased in CSF from AD patients compared with those in age-matched controls, as quantified by microarrays and qRT-PCR (Alexandrov et al., 2012; Lukiw et al., 2012). Currently, urine is collected and analyzed for biomarker discovery and diagnostic purposes in clinical practice (Weber et al., 2010; Bryant et al., 2012). Exosomes can be purified from the urine using various methods such as differential ultracentrifugation (Alvarez et al., 2012), which is highly valuable for investigating whether miRNAs can be detected in the urine. However, it remains difficult to determine whether the alteration in miRNA levels in humans is a cause or consequence of the neurodegenerative process. Investigation of miRNA analysis profiles in AD animal models might help to solve this problem. Additionally, the potential to test miRNAs in biological body fluids may contribute to developing and promoting the discovery of specific biomarkers for neurodegenerative diseases such as AD.

## Development of a Brain-Derived Exosomal Biomarker for AD

Currently, a mixed population of exosomes from various types of cells can be separated from biological fluids by multiple techniques such as classically differential ultracentrifugation, immunomagnetic beads and size exclusion chromatography (Li et al., 2017; Yu et al., 2018). Moreover, exosomes have a lipid bilayer to protect their cargo, which is used downstream, from RNAse treatment to be confirmed whether the miRNAs/mRNA analyzed are inside the exosomes or not (Cheng et al., 2014). This mixed population of exosomes may be recognized by western blots or mass spectrometry using proteins that are involved in the formation process of ILVs (Lotvall et al., 2014) as mentioned and discussed at the beginning of this manuscript. It is worth noting that many of these markers are not exclusive to exosomes and it is necessary to further examine the characteristics of exosomes (Watt et al., 2011). In the CNS, investigating cells from the brain may afford insights into the mechanisms of brain diseases (Saeedi et al., 2019). Isolating neuronal exosomes from cells related to AD may bridge the gaps in knowledge of peripheral biomarkers and provide mechanistic insight to this disease. Recently, a precipitation/immunoaffinity system has been developed to isolate neuron-derived and astrocyte-derived exosomes from the blood of AD patients (Goetzl et al., 2018). Data from these studies suggest that BDEs from blood plasma and measurement of certain forms of tau in BDEs can be used as diagnostic and prognostic biomarkers for AD (Guix et al., 2018; Saeedi et al., 2019).

Enrichment of a specific neuron-derived population of exosomes permits monitoring of target cells of interest (Table 2). Collectively, although exosome transfer of Aβ seems to mainly occur in AD and can be exploited as a helpful biomarker of the disease course, development of additional exosome biomarkers could contribute to a more accurate diagnosis of AD and discovering further close connections between the marker and mechanisms of the early stage of AD as well as other neurodegenerative diseases.

**TABLE 2:** The biomarker of different neural derived exosomes (NDEs).

**Exosomes isolated from different cell types**	**Biomarker**	**Function**
Cortical neurons-derived exosomes; immature and mature hippocampal neurons exosomes	The GluR2/3 subunits of glutamate receptors	Neuronal markers and play key roles in virtually all excitatory neurotransmission in the brain Faure et al., 2006.
	L1 cell adhesion molecule (L1CAM)	Neuronal markers, cell adhesion molecule with an important role in the development of the nervous system Lachenal et al., 2011.
Microglia-derived exosomes	Ionized calcium binding adaptor molecule 1 (Iba1)	A microglia/macrophage-specific calcium-binding protein with actin-bundling activity that participates in membrane ruffling and phagocytosis in activated microglia Raffo-Romero et al., 2018.
Astrocytic-derived exosomes	Glutamine aspartate transporter (GLAST)	Selective markers of astrocytic plasma membranes Raffo-Romero et al., 2018.
	Glial fibrillary acidic protein (GFAP)	A specific marker for astrocytes; the astrocytic cytoskeleton Goetzl et al., 2016b.
	Glutamine synthetase (GS)	Astrocyte marker, it catalyzes the production of glutamine and 4-aminobutanoate Goetzl et al., 2016b.
Oligodendrocytes-derived exosomes	Myelin proteolipid protein (PLP)	Oligodendrocytes marker, it is the major myelin protein from the central nervous system. It plays an important role in the formation or maintenance of the multilamellar structure of myelin Kramer-Albers et al., 2007.
	2′, 3′-cyclic nucleotide 3′-phosphodiesterase (CNP)	Oligodendrocytes marker, it belongs to the cyclic nucleotide phosphodiesterase family Kramer-Albers et al., 2007.

## Exosomes as Novel AD Therapeutics

The BBB is a continuous endothelial membrane within brain microvessels and is sheathed by mural vascular cells and perivascular astrocyte end-feet, which seal the cell−to−cell contacts to prevent the transmission of potentially toxic compounds between the brain and the blood (Matsumoto et al., 2017). In addition to transmembrane diffusion of small (<400 Da) lipid-soluble molecules, the BBB permits selective transport of some compounds into and out of the brain (Sanchez-Covarrubias et al., 2014).

Exosomes have an inherent ability to cross the BBB, and because their properties remain active in the brain, they are ideal drug delivery vehicles. This BBB-penetrating capacity, which was first reported by Alvarez-Erviti et al. (2011), resulted in effective delivery of small-molecule drugs to the brain through systemic injection of naked exosomes in mice, leading to promotion of drug-mediated biological responses (Zhuang et al., 2011). Later studies have been successful in transmitting exosomes through intranasal injection into the mouse brain (Aliotta et al., 2007). Recently, a study using rats identified that the fluorescently tagged forebrain astrocyte protein aldolase C was selectively expressed in brain tissue and could be recovered in exosomes in the blood (Ratajczak et al., 2006; Gomez-Molina et al., 2019). This study affords evidence of communication mediated by exosomes from the brain to the rest of the body (Ratajczak et al., 2006; Gomez-Molina et al., 2019). Evidence from these studies illustrates that exosomes can cross the BBB in a bi-directional manner; however, their method of accurately crossing the BBB remains unclear and requires further study.

In contrast, while exosomes may play a role in the spreading of AD, some studies have shown a positive effect of introducing non-pathogenic exosomes to change disease duration and progression (Table 3). In animal studies, this therapeutic effect was found when exosomes from young mice were observed to significantly downregulate aging-associated signaling molecules such as IGF1R and upregulate telomerase-related genes such as *Men1, Mre11a, Tep1, Terf2, Tert*, and *Tnks* in aged mice (Lee et al., 2018). Furthermore, exosomes injected into the brain of transgenic mouse models of AD can help to decrease toxic oligomers and fibrils in a microglial-dependent manner following intracerebral administration, contributing to the clearance of Aβ *in vivo* (Yuyama et al., 2012, 2014; Yuyama and Igarashi, 2017). Other researchers have suggested that mesenchymal stromal-derived exosomes may have a therapeutic effect *in vivo* on the advancement of neurovascular plasticity in other neurodegenerative diseases such as stroke (Xin et al., 2013a).

**TABLE 3:** Exosome administration for the treatment of AD and other neurological disorders.

**Disease**	**Animal model**	**Source**	**Administration**				**Proposed mechanism and results**	**References**
			Concentration (Total amount)	Route	Period (time)	Sampling/Sacrifice		
	APP^sweInd^	N2a cells	2 mg / mL, 0.25 μL/h (168 μg)	Dentate gyrus	14 days (continuously)	14 days after surgery	Aβ clearence. Aβ level ↓; Amyloid deposit ↓; Synaptotoxicity ↓	Yuyama et al., 2014, 2015
	APP^sweInd^	Plateletfreeplasma	3 μg / 3 μL (3 μg)	Dentate gyrus	Single injection	3 days / 20 days after injection	Co-localization (exosome and Aβ)	Zheng et al., 2017
	hiPSCs Injected mice	Tau mutation hiPSCs	0.5 μg / 2 μL (0.5 μg)	Hippocampus	Single injection	1 m / 2 m after injection	Tau propagation. Neurodegeneration ↑	Winston et al., 2019
	5XFAD pups	Astrocyte	NA	Brain	Single injection	48 h after injection	Aβ plaque ↓ by exosome ↓. nSMase2 ↓; exosome ↓; Aβ plaque ↓	Dinkins et al., 2014
	Aβ-derived diffusible ligands injected mice	N2a cells human CSF	4 μg / 5 μL 4 μg	I.C.V.	Single injection	NA	Synaptic plasticity. LTP↑, Aβ action ↓	An et al., 2013
	AD mice	hUmbilical cord MSCs	30 μg / 0.1 mL 120 μg	I.V.	2 month (biweekly injection)	1 m after injection	Neuron inflammation↓. Aβ deposit ↓; activation of microglia ↓; pro-inflammatory levels ↑; anti-inflamatory cytokines ↓	Ding et al., 2018
	APP^sweInd^	(hypoxia) PC-MSCs	150 μg / 80 μL 1200 μg	I.V.	4 month (biweekly injection)	5 h after injection	Neuroprotection and Immunomodulation. Plaque deposition ↓; Aβ level ↓; activation of astrocytes ↓; activation of microglia ↓; TNF-α, IL-1β↓; IL-4 ↑	Cui et al., 2018
Stroke	MCAo, rat	MSCs	3 × 10^6^ / mL 3 × 10^6^	I.V.	24 h after surgery (single injection)	14 days after surgery	White matter repair	Cui et al., 2018
	MCAo, rat	MSCs	100 μg / 0.5 ml 100 μg	I.V.	24 h after surgery (single injection)	28 days after surgery	White matter repair	Xin et al., 2013a
	MCAo, rat	MSCs	100 μg/0.5 mL 100 μg	I.V.	24 h after surgery (single injection)	28 days after surgery	White matter repair	Xin et al., 2017
	Embolism, mouse	NSC EV	NA	I.V.	2 / 14 / 38 h after surgery (triple injection)	96 h after surgery	Immune modulation	Webb et al., 2018b
	MCAo, pig	NSC EV	NA	I.V.	2 / 14 / 24 h after surgery (triple injection)	1 / 84 days after surgery	Reduction in edema	Webb et al., 2018a
	ICH, rat	MSCs	NA	I.V.	12 h after surgery (single injection)	2 / 7 / 28 days after injection	Immunosuppression	Otero-Ortega et al., 2018
	Rat	MSCs	NA	NA	NA	NA	White matter remodeling	Buller et al., 2016
TBI	CCI, rat	hMSCs	100 μg / 0.5 ml 100 μg	I.V.	12 h after surgery (single injection)	35 days after surgery	Angiogenesis and neurogenesis	Zhang et al., 2017
	ICH, rat	MSCs	3 × 10^6^ MSCs 3 × 10^6^	I.V.	12 h after surgery (single injection)	12 days after surgery	Angiogenesis and neurogenesis	Kim et al., 2016
	TBI, swine	MSCs	1 × 10^15^; 1 × 10^13^	I.V.	6 h / 1 / 5 / 9 / 13 days	30 days after surgery	Neuroprotection	Williams et al., 2019
Fetal hypoxia	OCD singleton fetuses	MSCs	2.0 × 10^7^ cell; 4.0 × 10^7^	I.V.	1 h / 4 days (2)	7 days after surgery	Neuroprotection	Williams et al., 2019
ICH	ICH, rat	MSCs	100 μg protein I.V., 200 μg	I.V.	24 h after surgery (single injection)	28 days after surgery	Neurovascular and white matter remodeling.	Han et al., 2018
	ICH, rat	MSCs	100 μg protein I.V., 100 μg	I.V.	24 h after surgery (single injection)	28 days after surgery	White matter repair	Otero-Ortega et al., 2018
Focal ischemia	Photo thrombosis, mouse	BM-MSCs	NA	I.V.	24 h after surgery (single injection)	2 h after injection	Neurogenesis	Yang et al., 2017
ASD	BTBR T+tf/J mouse	hMSCs	3.81 × 10^8^ particles/5 μL 1.9 ×10^9^	I.N.	12 days (every other day injection)	Behavior test	Social interaction	Perets et al., 2018
Inflammation	C57BL/6j mice	EL-4 T cell	2 μg / 2 μL 10 μg / 10 μL	I.N.	10 min (every 2 min injection)	After administration	Anti-inflammation	Zhuang et al., 2011
Status epileoticus	C57BL/6J mice	MSCs	∼ 5 μg 30 μg / 150 μL	I.N.	18 h (every 5 min)	4 days after administration	Neurogenesis and memory dysfunction	Long et al., 2017
Bacterial infection	C57BL/6J mice	BMDCs	25 μg / 30 μL 75 μg / 90 μL	I.N.	2 week (three doses)	2 week after immunization	Macrophage and dendritic cell activation	Giri et al., 2010
	C57BL/6J mice	Bone marrow cells	25 μg / 30 μL 75 μg / 90 μL	I.N.	2 week (three doses)	2 week after dose	Potential mechanism for antigen cross-priming	Giri and Schorey, 2008

*APP^*swelnd*^, mice expressing the human APP bearing the Swedish and Indiana (KM670/671NL, V717F) mutations (J20); ASD, autism spectrum disorders; BM-MSCs, bone marrow mesenchymal stem cells; CCI, controlled cortical impact; CSF, cerebrospinal fluid; hiPSCs, human induced pluripotent stem cells; EV, extracellular vesicles; ICH, intracerebral hemorrhage; I.C.V., intracerebroventricularly infusion; IL, interleukin; I.N., intranasal administration; I.V., intravenous injection; LTP, Long-term potentiation; MCAo, middle cerebral artery occlusion; MSCs, mesenchymal stem cells; NA, not applicable; NSC, Neural stem cell; OCD, occluder; PC-MSCs, preconditioned mesenchymal stem cells; TBI, traumatic brain injury; TNF-α.*

Additionally, directed exosomal transmission systems for precision nanomedicine have attracted extensive interest across the fields of pharmaceutical sciences, molecular cell biology and nanoengineering (Zhu et al., 2018). Exosomes are also a promising type of novel drug delivery vehicle because of their ability to cross the BBB and shield their cargo from enzymatic and chemical degradation. Recent developments regarding nanoengineering using targeted exosomes for therapeutic purposes have been conducted by researchers, for example, Xin et al. (2013b), Tran et al. (2019).

Introducing exogenous exosomes into the CNS because they can effectively cross the BBB is a potentially novel strategy for AD therapies (Chen et al., 2016), and their innate secretion of enzymes could be beneficial for degrading toxic fibrils (Katsuda et al., 2015). The field of EV research is still at the initial stage in the CNS and yet improved therapeutic applications are already being developed.

## Opportunities and Challenges

Quantifying changes in EV cargo would be extremely difficult because of the lack of unique region-specific markers for circulating exosomes and the inaccessibility of specific brain tissue EVs from living patients. Interesting lines of research have examined both the induction of AD using pathogenic EVs and the sequestration of toxic plaques using exogenous healthy EVs.

It difficult to distinguish among EV types simply on the basis of protein markers or size alone. To better interpret and replicate the experimental results of exosome studies, combined exosome isolation methods as well as improved techniques for accurate purification and characterization are recommended. In addition, a crowdsourcing knowledgebase currently allows researchers in the EV field to track the latest EV biology and methodology (Lee et al., 2019).

In recent years, research has been focused on BDEs to attempt to solve questions of brain-associated disorders using blood biopsies. Exosomes isolated from plasma were used to enrich BDEs (Sun et al., 2017; Saeedi et al., 2019). This study demonstrated that both the number of neural-derived exosomes as well as the expression of Aβ, neurofilament light chain, and high-mobility group box 1 potentially act as biomarkers of neuropsychological impairment in HIV (Sun et al., 2017). BDEs from plasma have also been tested in a pilot study to examine protein biomarkers for patients with major depressive disorder (Kuwano et al., 2018). Moreover, in military personal with mild traumatic brain injuries, compared with controls, the levels of tau, Aβ42, and IL-10 deposited by BDEs were elevated (Kuwano et al., 2018). Cargo proteins and miRNA from astrocytic-derived exosomes have been analyzed to obtain mechanistic insight into AD (Goetzl et al., 2016b). Additionally, other cell-derived exosomes have also been researched for other brain-related disorders. The ability to access BDEs in plasma and other biological body fluids such as CSF and urine shows potential for clinical use in treating nervous system disorders.

## Conclusion

Although the domain of exosome investigation, especially BDEs, remains relatively novel, attractive evidence from other fields demonstrates that investigation of exosomes can afford insight into the disease mechanisms and processes associated with AD and treatment responses. Currently, increased research on exosomes has focused on biomarkers of the course of AD and their ability to mediate cell-to-cell communication in the nervous system. However, additional work is needed with respect to the mechanisms of bi-directional transport of cargo-carrying exosomes across the BBB, the alterations in the number or size of exosomes secreted, changes in cargo constituents, and identification of differences in specific cell types. Meantime, it is necessary to take into consideration that several preparations may contain another type of EVs given the procedure used to obtain them. Exosomes derived from cells in the CNS have tremendous biomarker potential because they may reverse physiological changes in nervous system disorders, and these changes can be tested in the periphery.

## Author Contributions

CQ and ZS designed the project. ZS, YX, WD, LZ, HZ, YH, PY, YQ, and WZ performed a majority of writing the manuscript. All authors reviewed and revised the manuscript.

## Conflict of Interest

The authors declare that the research was conducted in the absence of any commercial or financial relationships that could be construed as a potential conflict of interest.

## Abbreviations

α -SYN: α -synuclein
A β: amyloid β -protein
AD: Alzheimer’s disease
ALIX: ALG-2-interacting protein X
AMPA: α -amino-3-hydroxy-5-methyl-4-isoxazolepropionic acid
Apo-E: apolipoprotein E
APP: amyloid precursor protein
ARF6: ADP ribosylation factor 6
BACE1: beta-secretase 1
BBB: blood brain barrier
BDNF: brain derived neurotrophic factor
BDEs: brain derived exosomes
BIN1: Bridging I Ntegrator 1
cAMP: cyclic adenosine monophosphate
CD: Cluster of differentiation
CNS: central nervous system
CREB1: cAMP responsive element binding protein 1
CSF: cerebrospinal fluid
CT: computed tomography
ESCRT: the endosomal sorting complex required for transport
ECG: Electrocardiography
EE: early endosome
EGFR: epidermal growth factor receptor
EVs: extracellular vesicles
gDNA: genomic DNA
GAP43: growth associated protein 43
HSP: heat shock protein
IRS1: insulin receptor substrate 1
Iba1: ionized calcium-binding adapter molecule 1
IGF1: insulin-like growth factor 1
IL-1 β: interleukin 1 beta
ILVs: intraluminal vesicles
LE: late endosome
L1CAM: neuronal L1 cell adhesion molecule
LRP6: LDL receptor related protein
MCI: mild cognitive impairment
miRNA: micro ribonucleic acid
MMPs: matrix metalloproteinase regulators
MRI: magnetic resonance imaging
mRNA: messenger ribonucleic acid
MS: multiple sclerosis
MSCs: mesenchymal stromal cells
MVBs: multivesicular bodies
MVEs: multivesicular endosomes
Neuro-2a: Murine neuroblastoma
nSMase2: neutral sphingomyelinase-2
NSCs: neural stem cells
NTA: nanoparticle tracking analysis
PD: Parkinson’s disease
PrP: prion protein
p-S 396-tau: Tau phosphorylation at serine 396
p-T 181-tau: Tau phosphorylation at threonine 181
qPCR real-time: quantitative polymerase chain reaction
REST: re1 silencing transcription factor
S1P: sphingosine-1-phosphate
S1PR: S1P receptor
siRNA: small interfering ribonucleic acid
SMS2: sphingomyelin synthase 2
sMVBs: secretory multivesicular bodies
TGF β: transforming growth factor beta
TSG101: tumor susceptibility gene 101 protein.
